# Association of initial COVID‐19 vaccine hesitancy with subsequent vaccination among pregnant and postpartum individuals

**DOI:** 10.1111/1471-0528.17189

**Published:** 2022-05-19

**Authors:** Katherine Germann, Miranda K. Kiefer, Kara M. Rood, Rebecca Mehl, Jiqiang Wu, Radhika Pandit, Courtney D. Lynch, Mark B. Landon, William A. Grobman, Maged M. Costantine, Kartik K. Venkatesh

**Affiliations:** ^1^ College of Medicine The Ohio State University College of Medicine Columbus Ohio USA; ^2^ Division of Maternal–Fetal Medicine, Department of Obstetrics and Gynecology The Ohio State University College of Medicine Columbus Ohio USA; ^3^ Department of Obstetrics and Gynecology The Ohio State University College of Medicine Columbus Ohio USA

**Keywords:** COVID‐19, maternal immunization, pregnancy, vaccination, vaccine hesitancy, vaccine uptake

## Abstract

**Objective:**

To examine the association between initial COVID‐19 vaccine hesitancy and subsequent vaccination among pregnant and postpartum individuals.

**Design:**

Prospective cohort.

**Setting:**

A Midwestern tertiary‐care academic medical center. Individuals completed a baseline vaccine hesitancy assessment from 22 March 2021 to 2 April 2021, with subsequent ascertainment of vaccination status at 3–6 months follow‐up.

**Methods:**

We used multivariable Poisson regression to estimate the relative risk of vaccination by baseline vaccine hesitancy status, and then characteristics associated with vaccination.

**Main outcome measures:**

Self‐report of COVID‐19 vaccination, and secondarily, consideration of COVID‐19 vaccination among those not vaccinated.

**Results:**

Of 456 individuals (93% pregnant, 7% postpartum) initially surveyed, 290 individuals (64%; 23% pregnant, 77% postpartum) provided subsequent vaccination status (median = 17 weeks). Of these 290 individuals, 40% (116/290) reported COVID‐19 vaccine hesitancy upon enrolment, of whom 52% reported subsequent vaccination at follow‐up. Few individuals transitioned during the study period from vaccine hesitant to vaccinated (10%); in comparison, 80% of those who were not vaccine hesitant were vaccinated at follow‐up (aRR 0.19, 95% CI 0.11–0.33). Among those who remained unvaccinated at follow‐up, 38% who were vaccine hesitant at baseline were considering vaccination, compared with 71% who were not vaccine hesitant (aRR 0.48, 95% CI 0.33–0.67). Individuals who were older, parous, employed and of higher educational attainment were more likely to be vaccinated, and those who identified as non‐Hispanic black, were Medicaid beneficiaries, and were still pregnant at follow‐up were less likely to be vaccinated.

**Conclusions:**

COVID‐19 vaccine hesitancy persisted over time in the peripartum period, and few individuals who reported hesitancy at baseline were later vaccinated. Interventions that address vaccine hesitancy in pregnancy are needed.

## Tweetable abstract

COVID‐19 vaccination hesitancy among pregnant and postpartum individuals persists over time, and few of the individuals who reported hesitancy were later vaccinated.

## 1 INTRODUCTION

Vaccine hesitancy, defined as uncertainty or refusal of a vaccine despite the availability of vaccine services, is a critical barrier to achieving high vaccination coverage against the SARS‐CoV‐2 virus in the peripartum period.^1^, ^2^ Vaccination in pregnancy for influenza and pertussis is common practice to prevent maternal and neonatal morbidity.^3^ Over the past 12 months since vaccines against the SARS‐CoV‐2 virus first became available, studies have demonstrated a higher frequency of vaccine hesitancy and a lower frequency of vaccination among pregnant individuals, compared with their non‐pregnant counterparts of reproductive age.^4^, ^5^, ^6^ This disparity in vaccination is of public health importance because pregnant individuals with COVID‐19 are at a higher risk of maternal and obstetric complications compared with those without COVID‐19.^7^, ^8^, ^9^, ^10^

The relationship between initial COVID‐19 vaccine hesitancy in pregnancy and subsequent vaccination in the peripartum period is unclear, and recent data from outside of pregnancy suggest that COVID‐19 vaccine hesitancy may decrease over time.^11^ It is possible that COVID‐19 vaccine hesitancy in pregnancy may change over time as a result of evolving knowledge about the vaccine, non‐pregnant status, and changes in vaccine‐related attitudes and beliefs.^1^ Although pregnant and lactating individuals were excluded from the first COVID‐19 vaccine trials, increasing observational data have demonstrated the safety of these vaccines with no increased risk of adverse perinatal outcomes at birth in the absence of longer‐term safety data.^12^, ^13^, ^14^ In response, although obstetrician gynaecologist (OB/GYN) professional societies initially emphasised person‐centred decision making, in light of limited data, they have since recommended vaccination, as the benefits exceeded the risks.^15^, ^16^, ^17^ Finally, increasing knowledge of the risks of COVID‐19 in pregnancy,^7^ as well as the presence of more transmissible variants,^18^ may also impact vaccine hesitancy and vaccination.

Our objective was to examine the association between initial COVID‐19 vaccine hesitancy and subsequent vaccination in the peripartum period.

## 2 METHODS

### 2.1 Study setting and participants

We conducted a follow‐up survey of vaccination status for 6 months after an initial cross‐sectional assessment of COVID‐19 vaccine hesitancy among pregnant or postpartum individuals receiving prenatal and postpartum care at a Midwestern academic tertiary‐care centre in the USA. As previously described,^4^ we initially enrolled pregnant and postpartum individuals from 22 March 2021 to 2 April 2021 (hereafter referred to as baseline), which was concurrent with the period of initial eligibility of pregnant individuals for the COVID‐19 vaccine in Ohio.^19^ At follow‐up, we contacted all previously enrolled participants, starting at 3 months and ending at 6 months after the initial survey (with a median duration from enrolment to follow‐up of 17 weeks) from 29 June 2021 to 20 November 2021.

Inclusion criteria for the vaccine hesitancy survey were individuals aged ≥18 years who either had a confirmed intrauterine gestation or were <10 weeks postpartum, and who were receiving prenatal or postpartum care. Participants who expressed a desire for vaccination were provided with contact information for vaccination.

This study was approved by The Ohio State University Institutional Review Board (ref no. 2021H0023, 23 February 2021). Informed consent was obtained at both enrolment and follow‐up. We followed good practice in the conduct and reporting of this study, including the Enhancing the Quality and Transparency of Health Research (EQUATOR) Network and the Strengthening the Reporting of Observational Studies in Epidemiology (STROBE) reporting guidelines.^20^ Participants were not involved in the development of this study. This study was funded by the Care Innovation and Community Improvement Program at The Ohio State University.

### 2.2 Data collection

At baseline, we initially assessed participant sociodemographic characteristics and perceptions about vaccination, including the willingness to be vaccinated for COVID‐19, influenza and pertussis, prior exposure to vaccination, and barriers and facilitators to becoming vaccinated, using an in‐person survey instrument and electronic health record (EHR) data abstraction.^4^ These questions were adapted from the US Centers for Disease Control and Prevention adult internet panel survey to assess vaccination in pregnancy,^21^ the WHO Vaccine Hesitancy Determinants Matrix,^22^ and the ‘3 Cs’ model (complacency, convenience and confidence), as outlined by the WHO Vaccine Communications Working Group.^2^ The adapted survey instrument was not pretested.

At follow‐up, we administered a brief survey on vaccination status. We used their contact information at the delivery encounter, available from the electronic health record (EHR). If participants did not respond after two phone calls, they were sent an email message, and those who did not respond to the email then received a final phone call. An identical survey by email or telephone was administered in English and was designed to be completed in approximately 5 minutes. Vaccine status was assessed with the following three questions, ‘Have you been vaccinated for COVID‐19 since you completed a survey on COVID‐19 vaccination in pregnancy?’ (yes or no); and for those who did not report vaccination, ‘Do you plan to be vaccinated for COVID‐19 in the next 6 months?’ (yes, no, undecided) and ‘If you do not plan to be vaccinated, please provide the primary reason to not be vaccinated?’ (free response). Vaccination was defined as self‐reported receipt of at least one dose of any available COVID‐19 vaccine.

### 2.3 Exposures, outcomes and covariates

The primary exposure was COVID‐19 vaccine hesitancy as defined by the World Health Organization (WHO) Strategic Advisory Group of Experts (SAGE) on Vaccine Hesitancy, which was uncertainty or refusal of vaccination despite the availability of vaccination services.^23^

The primary outcome was self‐reported COVID‐19 vaccination status at follow‐up. Vaccination was defined as self‐reported receipt of at least one dose of any available COVID‐19 vaccination. Secondarily, we assessed whether an individual who had not been vaccinated was considering having a vaccination in the next 6 months after the follow‐up survey. ‘Considering vaccination’ was defined as those who reported they were either planning to be vaccinated or were undecided about vaccination (i.e. vaccination was still possible). The comparison group for both outcomes was those who were not and did not plan to be vaccinated (i.e. vaccinated at follow‐up was unlikely). We also assessed reasons for not planning for vaccination among the subset for whom vaccination at follow‐up was unlikely. A core outcome set was not used.

Confounding variables were selected for inclusion based on a directed acyclic graph (DAG) and based on prior studies of seasonal influenza vaccine hesitancy in pregnancy.^10^, ^21^

Models adjusted for age (continuous), self‐reported race and ethnicity (non‐Hispanic white, non‐Hispanic black, Hispanic, Asian and Other), parity (0, 1, 2 or more), trimester of pregnancy (first, second, third, fourth/postpartum) and chronic comorbid conditions (0, 1, 2 or more). In addition, when assessing the association between baseline vaccine hesitancy and subsequent vaccination status, we also adjusted for time from baseline to follow‐up assessment in weeks (continuous) and pregnant status at follow‐up (pregnant, postpartum). Race and ethnicity were self‐reported by the participants and were categorised using the criteria outlined by the US National Vital Statistics System. The use of the terms ‘race’ and ‘ethnicity’ recognizes these terms as social constructs and does not presuppose a biological construct.

### 2.4 Statistical analysis

We plotted alluvial or Sankey diagrams to demonstrate the path of individuals from baseline hesitancy status to follow‐up vaccination status, with the widths corresponding to the proportions observed. We examined the association between baseline vaccine hesitancy and subsequent vaccination at follow‐up. We then examined the association between baseline vaccine hesitancy and consideration of COVID‐19 vaccination among those not yet vaccinated. We explored baseline patient characteristics and perceptions about vaccination associated with both vaccination and considering vaccination at follow‐up. To conduct the above analyses, we used modified Poison regression with robust standard errors to estimate unadjusted relative risk and adjusted relative risk (RR and aRR). All statistical analyses were performed using Stata 16.1 (StataCorp, College Station, TX, USA) and R 4.1.2 (R Project for Statistical Computing).

## 3 RESULTS

### 3.1 Patient characteristics

Of the original 456 individuals who completed the baseline cross‐sectional survey (Figure S1), 335 were successfully contacted (73%) at follow‐up, 290 (64%) of whom consented to participate. Those who were not enrolled in the follow‐up, whether they declined or were unable to be contacted, were more likely to be of older age, to identify as non‐Hispanic black and to be a Medicaid beneficiary, and were less likely to have a college education (*p* < 0.01 for all) (Appendix S1; Table S1). The frequency of baseline COVID‐19 vaccine hesitancy was lower among those who were enrolled at follow‐up compared with those who were not (40% vs. 58%; *p* < 0.001).

At initial enrolment, the majority of participating individuals (95%, *n* = 275) were pregnant, and of those pregnant, the mean gestational age was 18 weeks (SD: 8.41). Their mean age was 30 years (SD: 5.12), 23% were non‐Hispanic black, 40% were Medicaid beneficiaries, 23% had completed high school or less education, and 70% were currently employed (Table 1).

**TABLE 1:** COVID‐19 vaccination status and likelihood of future vaccination by baseline COVID‐19 vaccine hesitancy among pregnant and postpartum individuals (*n* = 290)

Characteristic	Vaccinated at follow‐up	Considering vaccination at follow‐up	Unlikely to be vaccinated at follow‐up	Vaccinated vs. not vaccinated	Considering vaccination vs. unlikely to be vaccinated
	*n* = 151	*n* = 65	*n* = 74	aRR (95% CI)	aRR (95% CI)^a^
At baseline					
Age, years, mean (SD)	30.9 (4.59)	29.8 (5.07)	28.2 (5.74)	**1.03 (1.01–1.05)**	**1.03 (1.00–1.07)**
Race and ethnicity					
Non‐Hispanic white	117 (77.5)	29 (44.6)	38 (51.4)	1.00	1.00
Non‐Hispanic black	14 (9.3)	26 (40.0)	28 (37.8)	**0.36 (0.22–0.59)**	1.14 (0.77–1.68)
Hispanic	5 (3.3)	5 (7.7)	6 (8.1)	0.61 (0.32–1.19)	1.31 (0.66–2.59)
Other	15 (9.9)	5 (7.7)	2 (2.7)	1.05 (0.80–1.40)	**1.70 (1.01–2.86)**
Education (*n* = 289)					
High school or less	11 (7.3)	20 (30.8)	35 (47.3)	1.00	1.00
Some college	15 (10.0)	17 (26.2)	18 (24.3)	1.68 (0.84–3.29)	1.19 (0.72–1.99)
Bachelor’s degree	70 (46.7)	19 (29.2)	14 (18.9)	**2.93 (1.63–5.25)**	1.30 (0.76–2.23)
Advanced degree	54 (36.0)	9 (13.9)	7 (9.5)	**3.29 (1.81–5.97)**	1.48 (0.83–2.63)
Parity, ≥1	84 (55.6)	42 (64.6)	56 (75.7)	**0.73 (0.60–0.90)**	0.71 (0.50–1.02)
Employed	122 (80.8)	45 (69.2)	37 (50.0)	**1.59 (1.16–2.16)**	**1.58 (1.04–2.38)**
Health insurance, Medicaid	30 (19.9)	33 (50.8)	52 (70.3)	**0.47 (0.33–0.66)**	**0.67 (0.46–0.99)**
Substance use, current					
Tobacco	5 (3.3)	5 (7.7)	14 (18.9)	0.52 (0.24–1.13)	0.51 (0.24–1.09)
Drugs	7 (4.6)	7 (10.8)	11 (14.9)	0.61 (0.33–1.11)	0.84 (0.46–1.54)
Gestational age, weeks, mean (SD) at baseline	18.5 (8.20)	18.8 (8.44)	17.8 (8.89)	–	–
First trimester	50 (33.1)	17 (26.2)	27 (36.5)	1.00	1.00
Second trimester	76 (50.3)	39 (60.0)	35 (47.3)	0.96 (0.76–1.20)	1.51 (0.99–2.31)
Third trimester	17 (11.3)	6 (9.2)	8 (10.8)	1.15 (0.85–1.56)	1.20 (0.58–2.49)
Fourth trimester (postpartum)	8 (5.3)	3 (4.6)	4 (5.4)	1.16 (0.67–2.03)	1.41 (0.53–3.72)
Body mass index, kg/m^2^, mean (SD)	30.1 (7.34)	32.2 (7.58)	33.0 (9.88)	0.99 (0.98–1.00)	0.99 (0.97–1.01)
Chronic comorbid conditions					
None	89 (58.9)	38 (58.5)	45 (60.8)	1.00	1.00
1	53 (35.1)	19 (29.2)	16 (21.6)	1.17 (0.95–1.43)	1.19 (0.81–1.76)
2 or more	9 (6.0)	8 (12.3)	13 (17.6)	0.65 (0.38–1.09)	0.78 (0.43–1.41)
Prior COVID‐19 infection (*n* = 288)	13 (8.7)	10 (15.4)	9 (12.2)	0.72 (0.48–1.07)	1.11 (0.69–1.77)
Prior household COVID‐19 exposure	19 (12.6)	7 (10.8)	12 (16.2)	0.92 (0.65–1.30)	0.69 (0.38–1.25)
Friend or family member has received COVID‐19 vaccine (*n* = 289)	144 (96.0)	44 (67.7)	42 (56.8)	**4.03 (1.83–8.86)**	1.28 (0.82–1.98)
Vaccination uptake					
Tdap in current pregnancy (*n* = 283)	128 (86.5)	46 (74.2)	43 (58.9)	**1.59 (1.11–2.28)**	1.47 (0.96–2.27)
Influenza vaccine in current year (*n* = 289)	128 (84.8)	30 (46.9)	23 (31.1)	**2.73 (1.87–3.97)**	**1.41 (1.00–1.99)**
Influenza vaccine last year	133 (88.1)	48 (73.9)	36 (48.7)	**2.10 (1.41–3.11)**	**2.00 (1.30–3.10)**
Vaccination discussion with OB/GYN provider					
Any infection in pregnancy (*n* = 286)	126 (84.6)	42 (66.7)	47 (63.5)	**1.55 (1.12–2.16)**	1.12 (0.77–1.64)
COVID‐19 (*n* = 287)	106 (70.7)	30 (47.6)	19 (25.7)	**1.55 (1.19–2.03)**	**1.71 (1.21–2.42)**
Concerned about contracting COVID‐19 and impact to self and pregnancy, 1 to 10, mean (SD) (*n* = 285)	6.9 (2.58)	5.4 (2.80)	4.1 (3.08)	**1.13 (1.09–1.18)**	**1.08 (1.02–1.15)**
Benefit of vaccination					
Tdap (*n* = 280)					
Mother	9 (6.1)	5 (7.9)	7 (10.0)	1.00	1.00
Baby	45 (30.6)	15 (23.8)	23 (32.9)	1.22 (0.77–1.92)	0.98 (0.43–2.21)
Both	93 (63.3)	43 (68.3)	40 (57.1)	1.21 (0.78–1.88)	1.23 (0.59–2.60)
Influenza (*n* = 284)					
Mother	19 (12.7)	17 (27.0)	20 (28.2)	1.00	1.00
Baby	1 (0.7)	6 (9.5)	2 (2.8)	0.32 (0.06–1.82)	1.73 (0.97–3.08)
Both	130 (86.7)	40 (63.5)	49 (69.0)	**1.76 (1.23–2.50)**	1.04 (0.70–1.57)
COVID‐19 (*n* = 269)					
Mother	29 (19.6)	15 (25.9)	25 (39.7)	1.00	1.00
Baby	2 (1.4)	3 (5.2)	2 (3.2)	0.70 (0.25–1.96)	2.13 (0.97–4.69)
Both	117 (79.1)	40 (69.0)	36 (57.1)	**1.46 (1.09–1.95)**	1.53 (0.98–2.39)
At follow‐up					
Pregnant	21 (13.9)	21 (32.3)	26 (35.1)	**0.54 (0.38–0.77)**	0.80 (0.53–1.22)
Time from baseline to follow‐up, weeks, median (IQR)	30 (18, 31)	18 (14, 18)	18 (14, 19)	**1.05 (1.04–1.06)**	0.98 (0.95–1.02)

*n* = 290 for vaccinated vs. not vaccinated; *n* = 139 for considering vaccination vs. unlikely to be vaccinated (subset who was not vaccinated). Results in bold reflect statistically significant finding (*p* < 0.05).

^a^ Adjusted model included the following baseline covariates: maternal age, parity, race, trimester of pregnancy and chronic comorbid conditions.

At follow‐up, 23% (*n* = 68) were still pregnant, and those who were pregnant were less likely to be vaccinated compared to those who were postpartum (14% vs. 35%; aRR: 0.54; 95% CI: 0.38, 0.77). The follow‐up period was from 3 to 6 months after completion of the initial survey and the median duration from baseline to follow‐up was 18 weeks (IQR: 17 to 31), and was longer for those who reported vaccination at follow‐up [median: 30 weeks (IQR: 18 to 31)] compared to those who were unlikely to be vaccinated [median: 18 weeks (IQR: 14 to 19) (aRR: 1.05; 95% CI: 1.04, 1.06). Pregnancy status and duration to follow‐up did not vary between those who were considering vaccination versus those for whom vaccination was unlikely.

### 3.2 Association between vaccine hesitancy and vaccination

**FIGURE 1:**
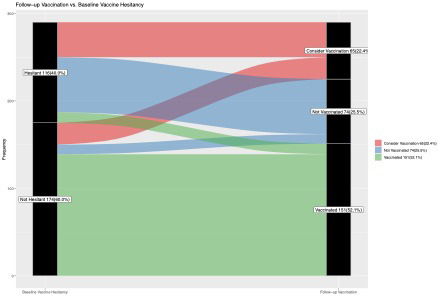
Alluvial plot paths from baseline hesitancy to follow‐up vaccination among pregnant and postpartum individuals. Alluvial plots show the path of individuals from baseline hesitancy status to follow‐up vaccination status, with the widths corresponding to the proportions observed

Of those followed‐up for 6 months, 40% (95% CI 34–46%) reported baseline COVID‐19 vaccine hesitancy at enrolment. At follow‐up, 52% (95% CI 46–58%) reported vaccination and 22% (95% CI 18–27%) were considering vaccination. As shown in the alluvial plot (Figure 1), few individuals transitioned from vaccine hesitant to vaccinated (10%, 95% CI 5–16%); in comparison, 80% (95% CI 74–86%) of those without vaccine hesitancy reported vaccination at follow‐up. Among those who were not vaccinated, 38% (95% CI 29–48%) with baseline vaccine hesitancy were considering vaccination, compared with 71% (55–87%) without baseline vaccine hesitancy.

In multivariable analysis, individuals who expressed COVID‐19 vaccine hesitancy at baseline were less likely to report subsequent vaccination, compared with individuals without vaccine hesitancy (aRR 0.19, 95% CI 0.11–0.33). If they remained unvaccinated at follow‐up, they were also less likely to be considering vaccination (aRR 0.48, 95% CI 0.33–0.67) (Table 2).

**TABLE 2:** Relative risk of COVID‐19 vaccination or considering vaccination by COVID‐19 vaccine hesitancy status at baseline

	RR (95% CI)	aRR (95% CI)^a^
Vaccinated vs. not vaccinated	**0.13 (0.08–0.22)**	**0.19 (0.11–0.33)**
Considering vaccination vs. unlikely to be vaccinated	**0.54 (0.39–0.74)**	**0.48 (0.33–0.67)**

*n* = 290 for vaccinated vs. not vaccinated; *n* = 139 for considering vaccination vs. unlikely to be vaccinated (subset who was not vaccinated). Results in bold reflect statistically significant findings (*p* < 0.05).

^a^ Adjusted model included the following covariates at baseline: age, parity, race, trimester of pregnancy and chronic comorbid conditions; and at follow‐up: pregnant status and weeks from baseline to follow‐up.

**FIGURE 2:**
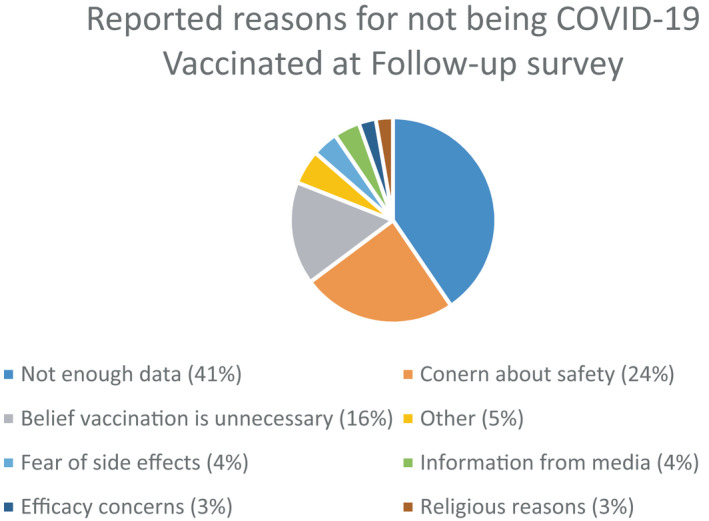
Frequency of reasons given for not being vaccinated at follow‐up

Among those at follow‐up who reported ‘vaccination was unlikely’, the most common (mutually exclusive) reasons for vaccine refusal were concerns about safety (24%), not enough data (41%) and the belief that vaccination is unnecessary (16%) (Figure 2).

### 3.3 Factors associated with vaccination

Looking at baseline demographic factors associated with COVID‐19 vaccination at follow‐up, individuals who identified as non‐Hispanic black, were parous and were Medicaid beneficiaries were less likely to report subsequent COVID‐19 vaccination (Table 2). Individuals who were of older age, had attained higher levels of education and were employed were more likely to report subsequent COVID‐19 vaccination. Chronic comorbid conditions, substance use and trimester at baseline assessment were not significantly associated with COVID‐19 vaccination.

Looking at other baseline factors associated with COVID‐19 vaccination at follow‐up, individuals who reported having a friend or family member who had received the COVID‐19 vaccine, who had or planned to receive the tetanus, diphtheria and pertussis (Tdap) vaccine in pregnancy, who had received the seasonal influenza vaccine in the current year or past year, who were more concerned about contracting COVID‐19 and its impact on self and pregnancy, and who perceived the benefit of COVID‐19 and influenza vaccinations for both the mother and the baby were more likely to report subsequent COVID‐19 vaccination (Table 2). Additionally, individuals who reported discussing any vaccinations and specifically COVID‐19 vaccinations in pregnancy with their OB/GYN healthcare provider were more likely to report COVID‐19 vaccination. Neither report of previous COVID‐19 infection nor prior household COVID‐19 exposure were associated with vaccination.

Examining the self‐report of considering vaccination among those who had not yet been vaccinated at follow‐up, individuals who were of older age, employed and had received the seasonal influenza vaccine in the current year were more likely to be considering vaccination, and those who were enrolled in public health insurance were less likely to be considering vaccination (Table 2). In addition, those who had discussed COVID‐19 vaccination with their OB/GYN healthcare provider and who were more concerned about contracting COVID‐19 because of its impact on self and pregnancy were also more likely to be considering vaccination.

## 4 DISCUSSION

### 4.1 Main findings

We found that COVID‐19 vaccine hesitancy at enrolment (93% pregnant) was associated with persistent hesitancy at follow‐up (23% pregnant) in the peripartum period. Only one‐tenth of those who were initially hesitant became vaccinated. COVID‐19 vaccine‐hesitant individuals were approximately 80% less likely to report consequent vaccination, compared with those who did not report COVID‐19 vaccine hesitancy. And among the individuals who remained unvaccinated at follow‐up, those who had reported initial COVID‐19 vaccine hesitancy were approximately 50% less likely to be considering vaccination, compared with those who had not reported vaccine hesitancy.

### 4.2 Strengths and limitations

A strength of this study is that we assessed the relationship between vaccine hesitancy and subsequent vaccination, given that vaccine hesitancy can be dynamic for the same individual through the peripartum period, as well as in the setting of rapidly evolving data about COVID‐19. Previous studies conducted in pregnancy have assessed vaccine hesitancy and vaccination at a single time point.

There are several study limitations to note. This study was a convenience sample of individuals enrolled in prenatal and postpartum care. For this follow‐up study, we were able to contact 73% and enrolled 64% of the original sample. Selection bias is possible as those who were not vaccinated may have been less likely to respond at follow‐up. And hence, vaccine uptake at follow‐up may have been overestimated. Those who did not enrol were more likely to report vaccine hesitancy and were more likely to identify as non‐Hispanic black, be Medicaid recipients and have lower education attainment, which are associated with lower rates of vaccination in pregnancy. Follow‐up was not a planned part of the original study protocol. We do not have follow‐up on all participants over 6 months. Those who reported vaccination were more likely to have been contacted later between baseline and 6 months, which could contribute to misclassification. We did not reassess factors associated with vaccine hesitancy at follow‐up, including exposure to COVID‐19 and breastfeeding status. Our outcome of vaccination status was based on self‐report rather than a biological test or a medical record, so misclassification through inaccurate self‐reporting is possible. When responding primarily by phone to a research assistant, it is possible that some participants may have been more likely to report vaccination or plan for vaccination versus being unlikely to be vaccinated. Self‐reported vaccine status for COVID‐19 outside of pregnancy has been shown to be congruent with biological tests (sensitivity 95%, specificity 99%).^11^ Additional information, including which vaccine was received and from where, was also not ascertained. Although such contextual information may also help determine the accuracy of self‐reported data, recent studies support the validity of self‐reported COVID‐19 results in the postpartum period.^32^ We did not assess other potential factors associated with vaccine uptake and hesitancy, including political and religious affiliation as well as social determinants of health using a standardised measure.^27^, ^33^ It is possible that many of the baseline characteristics assessed may have evolved over time, including vaccination discussions with OB/GYN healthcare providers and the offer and receipt of influenza and Tdap vaccinations. The survey instrument, although using questions and constructs from previously validated surveys, was not pre‐tested in our study population. Our results were from a single centre and may not be generalisable to all practice and regional settings. In addition, our results may evolve over time, as the follow‐up assessment ended the week before the announcement of the Omicron variant of the virus.

### 4.3 Interpretations

As individuals who were not vaccinated were less likely to participate in the follow‐up survey, our findings may overestimate vaccination in the peripartum period. A history of prior COVID‐19 infection was assessed at baseline (11%), and was not assessed at follow‐up, which could have also impacted the decision and timing of vaccine receipt. It is also possible that some individuals may have been vaccinated as a result of employment or government mandates. In addition, the pandemic timeline, including different variants, available vaccine safety data, and accompanying reports of morbidity and mortality in pregnancy, could have impacted the rate of vaccination. The baseline survey was conducted in the Spring of 2021, concurrent with state policies recommending vaccination in pregnancy, and the follow‐up survey was concluded before the Omicron variant was reported. Vaccination did not vary by trimester of pregnancy, and this should be studied further as patient and provider concerns about fetal safety may vary by trimester.

Our results differ from a recent study of >4000 respondents conducted from late 2020 to mid 2021 as part of a population‐based US cohort in which COVID‐19 vaccine hesitancy (baseline frequency 31%) decreased across a 6‐month follow‐up.^11^ In that study, more than one‐third of participants (37%) transitioned from vaccine hesitant to vaccinated. Although the period of follow‐up was similar between studies, those respondents were not restricted to the peripartum period. In pregnancy, prior cross‐sectional surveys conducted in the USA have demonstrated slightly higher vaccination rates than the current study, including 58% among patients enrolled in prenatal care in the North East,^5^ and 66% and 85%, respectively, among pregnant and breastfeeding healthcare workers in the Midwest.^6^ The low level of COVID‐19 vaccination observed in the current analysis is also consistent with data from other high‐income countries, including the UK (11%) and Israel (40%).^24^, ^25^ Vaccine hesitancy may also be prevalent among pregnant individuals in low‐ and middle‐income countries, as only 51% of pregnant individuals enrolled in the Global Network for Women’s and Children’s Health were able to report three or more measures associated with preventing COVID infection.^26^ Favourable attitudes toward COVID‐19 vaccines may be decreasing over time,^27^ and further data across regional settings is needed to understand how vaccination may evolve in the peripartum period.

In the current study, the two primary reasons for not being vaccinated at follow‐up were ‘concerns about safety’ and ‘not enough data’, which we have previously reported as the primary reasons for vaccine hesitancy at baseline.^4^ We also noted that individuals who reported vaccination for Tdap and influenza, discussed vaccination with their OB/GYN healthcare provider, who had a friend or family member who had been vaccinated for COVID‐19, and who expressed a belief that there was a benefit of vaccination for the baby and the mother were more likely to be vaccinated. We did not assess whether those who reported vaccine hesitancy at baseline and reported being unvaccinated at follow‐up received any intervening counselling or guidance from a healthcare provider encouraging vaccination. These findings with regards to the importance of provider recommendations and patient perceptions are consistent with prior studies assessing vaccination uptake in pregnancy.^4^, ^28^, ^29^ Further interventions that address communication to improve vaccine awareness and address safety data among pregnant and postpartum individuals and their healthcare providers are needed.^30^

Factors that affect COVID‐19 vaccination in the peripartum period include adverse social determinants of health, including minority race and ethnicity, low educational attainment and lack of access to quality health care.^31^ In the current study, we found many of the characteristics associated with a lower likelihood of vaccination, including non‐Hispanic black race/ethnicity, enrolment in public health insurance, lower educational status and lack of employment, were previously identified as predictors of vaccine hesitancy at baseline in this cohort.^4^ Individuals with these characteristics may face structural barriers to accessing healthcare services and adverse social determinants of health that are associated with a higher risk of complications from COVID‐19.^8^ Whether interventions that address social determinants of health affect COVID‐19 vaccination uptake in the peripartum period should be studied.

## 5 CONCLUSION

In conclusion, COVID‐19 vaccine hesitancy may persist in a sizeable proportion of individuals in the peripartum period. Given that the COVID‐19 pandemic is continuing and repeat vaccination is likely to be required,^34^ understanding vaccine hesitancy and vaccination over time in the peripartum period is a public health imperative.^1^ These findings suggest the durability of vaccine hesitancy over time, and the need for future interventions to increase COVID‐19 vaccination in the peripartum period.

## CONFLICT OF INTERESTS

None declared. Completed disclosure of interests form available to view online as supporting information.

## AUHTOR CONTRIBUTIONS

KKV, MKK, RM, KMR, and MMC designed the study. KG, RP, MKK, and RM conducted the study and and abstrated study data. JW, CDL, WAG, and KKV conducted all analyses. KG, MKK, CDL, MBL, WAG, MMC, and KKV interpetted study findings and wrote the manuscript.

## ETHICAL APPROVAL

This study was approved by The Ohio State University Institutional Review Board (ref. 2021H0023, 23 February 2021).

## Supporting information


Appendix S1

Data S1

## Data Availability

Data in de‐identiifed form of some variables can be available upon request to the corresponding author.
