# Supplementary material for: Association of initial COVID‐19 vaccine hesitancy with subsequent vaccination among pregnant and postpartum individuals
Source: BJOG. 2022 May 19;129(8):1352–60. doi: 10.1111/1471-0528.17189 (PMC9111102; doi:10.1111/1471-0528.17189)
Supplement: Supplementary file 1 — Appendix S1 [file BJO-129-1352-s001.docx]

| **Table S1. Comparison of selected baseline characteristics between non-enrolled versus enrolled participants at follow-up** | | | |
| --- | --- | --- | --- |
|  | **Not enrolled at follow-up^1^** | **Enrolled at follow-up** |  |
|  | **N=166** | **N=290** |  |
|  | **N (%)** | **N (%)** | **P value^2^** |
| **Age, mean (SD), years** | 28.0 (5.57) | 29.9 (5.10) | <0.001 |
| **Pregnancy status at baseline**  Pregnant  Postpartum | 160 (96.4)  6 (3.6) | 275 (94.8)  15 (5.2) | 0.45 |
| **Parity, mean (SD)** | 1.19 (1.31) | 1.17 (1.31) | 0.85 |
| **Race, self-reported**  White  Black  Hispanic  Other | 88 (53.0)  55 (33.1)  16 (9.6)  7 (4.2) | 183 (63.1)  67 (23.1)  17 (5.9)  23 (7.9) | 0.02 |
| **Insurance**  Private  Public | 67 (40.4)  99 (60.0) | 174 (60.0)  116 (40.0) | <0.001 |
| **Education (n=452)**  High school or less  Some college  Bachelors degree  Advanced degree | 63 (38.7)  39 (23.9)  43 (26.4)  18 (11.0) | 67 (23.2)  50 (17.3)  102 (35.3)  70 (24.2) | <0.001 |
| **Comorbid conditions**  0  1 or more | 47 (28.3)  119 (72.0) | 89 (30.7)  201 (69.3) | 0.59 |
| ^1^Includes participants who declined participation (n=45) or could not be contacted (n=121).  ^2^Chi square test used for categorical variables and Student T test for continuous variables. | | | |

**Figure S1. Flowchart of study participants**

Participants enrolled in baseline survey (N=456)

Participants approached for follow-up:

N=335 (73.5%)

Patients declined follow-up (n=45)

Enrolled patients

N=290 (86.6%)
